# Domestication and microbiome succession may drive pathogen spillover

**DOI:** 10.3389/fmicb.2023.1102337

**Published:** 2023-03-17

**Authors:** Péter Apari, Gábor Földvári

**Affiliations:** ^1^Institute of Evolution, Centre for Ecological Research, Budapest, Hungary; ^2^Centre for Eco-Epidemiology, National Laboratory for Health Security, Budapest, Hungary

**Keywords:** emerging infectious diseases, pathogen spillover, horizontal gene transfer, microbiome succession, virulence genes, disease ecology, prevention, domestication

## Abstract

Emerging infectious diseases have posed growing medical, social and economic threats to humanity. The biological background of pathogen spillover or host switch, however, still has to be clarified. Disease ecology finds pathogen spillovers frequently but struggles to explain at the molecular level. Contrarily, molecular biological traits of host-pathogen relationships with specific molecular binding mechanisms predict few spillovers. Here we aim to provide a synthetic explanation by arguing that domestication, horizontal gene transfer even between superkingdoms as well as gradual exchange of microbiome (microbiome succession) are essential in the whole scenario. We present a new perspective at the molecular level which can explain the observations of frequent pathogen spillover events at the ecological level. This proposed rationale is described in detail, along with supporting evidence from the peer-reviewed literature and suggestions for testing hypothesis validity. We also highlight the importance of systematic monitoring of virulence genes across taxonomical categories and in the whole biosphere as it helps prevent future epidemics and pandemics. We conclude that that the processes of domestication, horizontal gene transfer and microbial succession might be important mechanisms behind the many spillover events driven and accelerated by climate change, biodiversity loss and globalization.

## Introduction

Emerging infectious diseases (EIDs) have been of great medical, social and economic importance especially in the 21^st^ century. There has been great controversy regarding EIDs whether spillover (jumping from one host species to another) is an easy or difficult task for pathogens. The main problem is that many theoretical, conceptual and empirical studies support the notion that these events are frequent in nature but molecular biological traits of host-pathogen relationships with the existence of specific molecular binding mechanisms contradict to these arguments (Araujo et al., 2015; Nylin et al., 2018; Brooks et al., 2019; Wells and Clark, 2019). Although there is ample evidence that many host receptor families are redundant (e.g., Boeger et al., 2022) and there is evolutionary similarity across host taxa, there are still lot of unexplored molecular mechanisms. Two experiental studies suggest that host receptors providing conservative essential functions can evolve in a way that blocks effective binding of viruses without damaging these host functions (Elde et al., 2009; Koyanagi et al., 2010).

**Figure 1:**
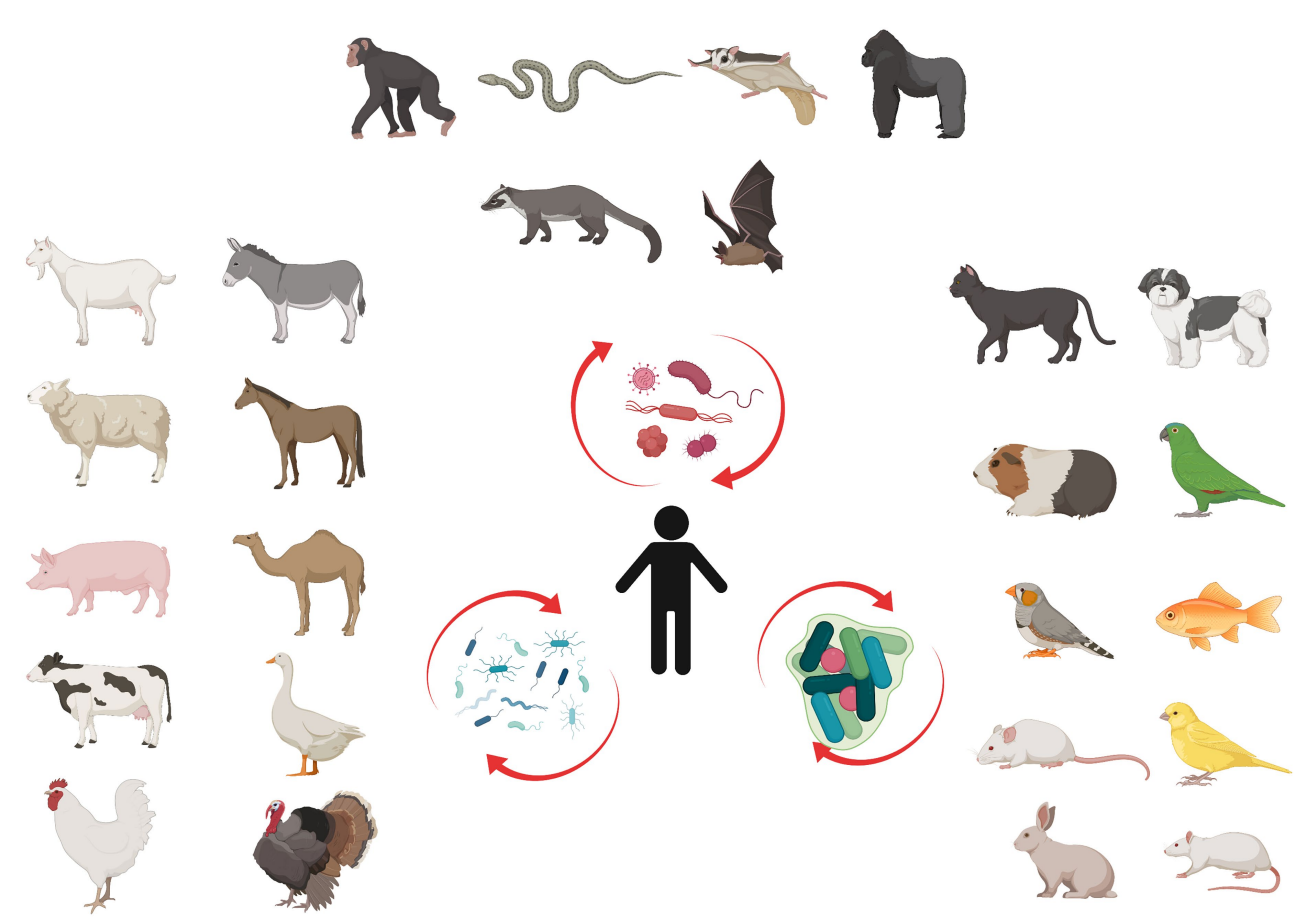
Microbiome diversity. Wildlife (above), domesticated livestock (left) and pet animals (right) have a constant influence on human microbiome. Initial changes in human microbiome trigger gradual modification called microbiome succession.

In this article we review current evidence at the molecular level to reconstruct a hypothetical bridge between molecular biology and ecology to support the high incidence theory of spillover in EIDs. We argue, that domestication of wild animals was a crucial step in the evolutionary history of human EIDs (Otte and Pica-Ciamarra, 2021). When humans started to domesticate farm and pet animals and got closer contact to bushmeat, the possibility of spillover of microorganisms between different species increased significantly (Reese et al., 2021). According to the main paradigm of the current scientific literature, the main factors making the gut microbiota of humans and their domesticated animals similar to each other are indirect interactions (Reese et al., 2021). However, we highlight the possibility that the changes in microbiome are not the result of indirect selection to a given diet but a direct incursion by foreign microorganisms *via* food consumption. Here we hypothesize that the first colonizing microbes initiated gradual changes in the human microbiome that have led to consecutive shifts in the microbiome community composition. As an analogy to ecological succession, i.e., the process of change in the species structure of plant, animal and fungus communities over time, we call this process microbiome succession (Figure 1).

We suggest four scenarios to support our hypothesis. (1) Bacteriophages and bacterial transformation events play an essential role in transporting virulence genes between host species that evolved for invasion and make it possible for foreign bacteria and viruses to enter new tissues and cells. In this process the possibility of gene transfer between superkingdoms (Archaea, Bacteria, Eukarya) and even viruses plays an essential role (Callier, 2019). (2) There is a gradual acceleration of microbiome exchange between host species by documented transfer of host phenotype with the newly arriving microbes (Bäckhed et al., 2004; Vrieze et al., 2012; Hu et al., 2013; Poutahidis et al., 2015). (3) The immunosuppression caused by one pathogen can facilitate the process to get another one (Devi et al., 2021). (4) Bacterial conjugation speeds up the exchange of microbiome between host organisms of different species by spreading genes that encode transferred phenotypes in a given host organism. With other words, we propose that gradual changes of the microbiota can increase their chance to infect or invade new hosts. This is enhanced by two processes. First, the newly acquired microbes will enable the colonization of the forthcoming new microbes with changes induced in the original microbiome. Second, horizontal gene transfer enhances transfer of virulence genes among the members of the microbiota. Overall, we present a new perspective at the molecular level which can explain the observations of frequent pathogen spillover events at the ecological level. Moreover, we also emphasize the importance and long-term effects of exchange of non-pathogenic microbiome in the current and future EID crisis.

## The role of transduction and bacterial transformation in emerging infections

Bacteriophages are in vast amounts in the biosphere, therefore we can find virus particles everywhere in soil, air and water (Hatfull, 2015). These microbes go through the animal and human body without problem (Huh et al., 2019). Only bacterial symbionts are infected with these viruses and they have two options (1) the lytic pathway in which bacterial cells are destroyed and (2) the lysogenic one where bacteriophage genome integrates into the host cell and enter into a dormant state (Huh et al., 2019). It is well-known that these microorganisms can transmit genetic elements, even virulence and antibiotic resistance genes between bacterial cells through the process called transduction (Kondo et al., 2021). According to molecular definition of the virulence genes they encode different kinds of virulence factors that are important for the pathogen’s successful replication and transmission to new host organisms (Alizon et al., 2009).

There are different adaptive functions for virulence factors like cell adhesion, immune evasion, freeing the hidden iron stores etc. From our perspective, the most interesting one is the pathogen’s capability to enter new cells and invade new tissues (Foster et al., 2014). This function can be essential for emerging pathogens to successfully infect and replicate in new hosts by breaking through the species barrier between them. As these molecular mechanism indicate, ecologist are often wrong when they try to ignore and dismiss molecular biology to solve the riddles of pathogen spillover to new host species (Brooks et al., 2019). But there is an issue in which ecologists are right and molecular biologist are wrong. The frequency of pathogen spillover is very high according to the research and experience of disease ecologists (Daszak et al., 2020; Grange et al., 2021) but this contradicts the concept of specific molecular mechanisms predicting the opposite tendency (Brooks et al., 2019). Our aim is to come up with a synthetic perspective integrating the disease ecological and molecular biological arguments in the question of pathogen spillover to reconcile them.

In times of global change and the large-scale spread of parasites across former geographic barriers, drawing conclusions about a pathogen’s host-shifting capacity using simple specificity indices may not be suitable to predict such events under novel conditions. Host specificity cannot be considered a fixed trait, as environmental conditions cause considerable variation in realized host specificity. On the other hand, the increasing recognition that local variation changes the suite of hosts to which a pathogen is exposed, and pre-existing capacity enables host shifting upon newly arising opportunities, has been raised by a number of recent studies that collectively contribute to a metatheory called the Stockholm Paradigm (Wells and Clark, 2019; Brooks et al., 2022). This recent evolutionary conceptual framework suggests that pathogens recognize evolutionary conserved host traits that are not species-specific. It argues, based on the concept of ecological fitting, that host changes are the result of changing conditions that bring pathogens into contact with susceptible hosts, with novel genetic variants arising in the new host after infection (Brooks et al., 2019). Molecular biology still wonders how key and lock nature of molecule binding can produce high number of pathogen jumps to new species (Brooks et al., 2019). With other words: how can these host recognition and host colonizing capabilities spread across taxa?

We can solve this problem with the help of horizontal gene transfer (Callier, 2019). Microorganisms containing virulence genes have the potential to adapt and successfully invade host cells and tissues (Foster et al., 2014). It is well-known that by the processes of transduction and bacterial transformation prokaryotes can exchange virulence genes and become detrimental to the health of the host organism (Gal-Mor and Finlay, 2006). This can explain the emergence of new bacterial pathogens in a host. But how do these virulence genes reach new host species and give the ability to new microorganisms to jump through the species barriers?

Bacteriophages are viruses which can infect bacteria and are extremely abundant in the whole biosphere (Hatfull, 2015). Intriguingly, these infectious particles are able to transmit genes between host organisms (Gal-Mor and Finlay, 2006; Callier, 2019). According to our current knowledge, they cannot replicate inside eukaryotic cells and tissues on their own but they effectively bind and replicate inside bacterial cells (Huh et al., 2019). Thus, bacteriophages are potentially less dangerous than viruses that infect eukaryotes, therefore the host organisms are more permissive to these microorganisms. There are published evidences for this, e.g., in patients, both endogenous phages in circulation and infusion of phages administered during lytic phage therapy are generally well tolerated with minimal inflammation (Popescu et al., 2021).

Moreover, the host can even protect itself from pathogenic bacteria by picking up vast amounts of bacteriophage particles (Dissanayake et al., 2019). This can explain how new bacterial pathogens emerge but give no information about those emerging viruses which are infecting eukaryotic cells. However, a recent paradigm shift revealed that genes of prokaryotic microorganisms can be horizontally transferred and successfully integrated and expressed in the genome of eukaryotic cells. *Wolbachia* spp. are endosymbiotic bacteria of many invertebrate hosts which can manipulate the sex ratio preferring females because they can only be transmitted vertically to the next generation by eggs (Kageyama et al., 2012). In an intriguing experiment, researchers observed sex bias towards females in many invertebrate insect hosts where no *Wolbachia* was detected. What mechanism could have led to such a bias? The answer was shocking because it has been revealed that the whole genome of *Wolbachia* has been integrated into the host genome as a new sex chromosome and controlled the sex ratio (Callier, 2019). This example shows that horizontal transfer of genes or even genomes is possible between distantly related organisms. We hypothesize that this mechanism might also lead to the transfer of virulence genes to enhance the colonization of new hosts for non-pathogenic organism. The horizontal spread of antibiotic resistance genes among bacterial strains or species is a well-known example for this mechanism.

In another exciting example, a toxin gene from a bacteriophage was transported to an insect host as a functional gene and protected the animal from its natural enemies. The exact molecular mechanism of this process is unknown (Callier, 2019). In the intestine and other organs of vertebrates we can find the mixture of bacteriophages, prokaryotes, eukaryotes and viruses that infect eukaryotic cells (Matijašić et al., 2020). Here, theoretically, by transformation events these microorganisms can exchange genes which can explain how virulence genes from bacteria and bacteriophages transferred to vertebrate viruses (and vice versa) enabling them to become emerging pathogens. Moreover, because intracellular bacteria can enter into host cells harboring different types of eukaryotic viruses, therefore they can exchange virulence genes with each other and bacteriophages can infect bacteria and pick up and transduce these genes (Malik et al., 2017).

The most frequent human pathogens which are affected in the process of spillover are RNA viruses (Zhang et al., 2020). Among these, retroviruses are able to integrate genetic elements into the host DNA (Peeters et al., 2010). In case of most RNA viruses, however, integration to the host DNA is also possible as shown by a recent study indicating that eukaryotic cells have their own polymerase enzymes with the capability of reverse transcription (Chandramouly et al., 2021). Horizontal gene transfer between phages and prokaryotes (Hall et al., 2020) is a highly frequent event. A new study systematically characterizing viral–eukaryotic gene exchange across eukaryotic and viral diversity identified thousands of transfers (Irwin et al., 2022).

## Microbiome succession facilitates pathogen emergence

The potential molecular mechanism for emergence of pathogens described above can explain the ecologically observed high incidence of spillovers of pathogenic microorganisms. However, there could be a parallel and partially overlapping mechanism if we consider the role of non-pathogenic micriobiota. Here we have to note that the pathogenic or mutualistic nature of microorganisms is a dynamic state depending on the nexus of many intrinsic and extrinsic interacting factors (Rózsa et al., 2015). These microorganisms can be transmitted vertically to the mammalian new-born by several mechanisms (Moossavi and Azad, 2020). It is interesting that the gut microbiota of adult female cows is very similar to the milk of these animals by which the microorganisms can be transmitted to young calves during the feeding process (Milani et al., 2019). Non-pathogenic or even mutualistic microbes, similarly to their pathogenic counterparts, can enter new host species and can permanently integrate into the new microbial environments (Milani et al., 2019). We hypothesize that this process is mutual, therefore the streaming of microbes between host organisms of different species has a vice versa nature.

We assume that it is not an easy task for the newcomers to establish themselves permanently into the new host. There is a so called colonization resistance which shields the existing network of microbes inside the host organism and protects it from the invasion of foreign or even pathogenic microorganisms (Pickard and Núñez, 2019). Interestingly, in this situation the nutritional conditions and the host’s genetic background is less important compared to the characteristics of the network of microbiome composition (Jones et al., 2022). A good example for the above-mentioned process could be the spreading of lactase persistence in the human population worldwide after the successful domestication of dairy animals (Ségurel and Bon, 2017).

In the Eurasian population there is only one dominant allele for lactose digesting enzyme while in the African population genetic polymorphism is the typical situation (Ségurel and Bon, 2017). The hypothesis that the expression of the gene encoding lactose digesting enzyme persists in adulthood because of domestication of wild animals and shift to agriculture is controversial (Ségurel and Bon, 2017). If we look at the worldwide big picture the genetic pattern correlates well with pastoral and agricultural habits but there are problems because exceptional cases exist. There are human populations, e.g., in Mongolia where the frequency of these genes is relatively low but the capability of digesting lactose is a unique characteristics of the whole community (Ségurel and Bon, 2017).

**Figure 2:**
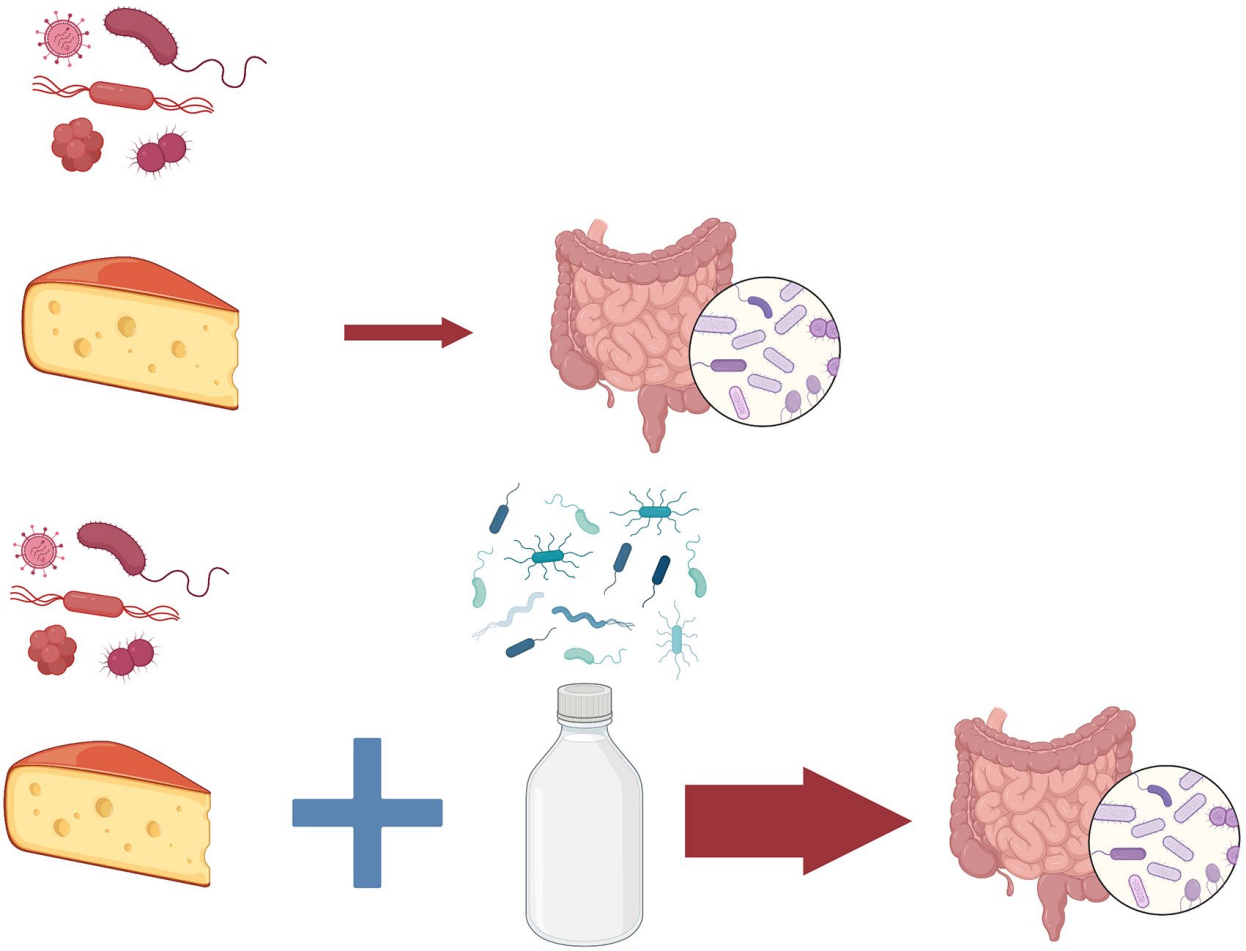
Microbiome succession. In an experiment (Milani et al., 2019) human participants consumed parmesan cheese containing *Bifidobacterium mongoliensis* a typical gut microorganism from dairy cow (above). The bacteria established themselves in the human gut and divided without complications even weeks later. When participants consumed milk with cheese at the same time, the fixation of the bacteria in the new intestinal environment was more successful (below) due to the changes in the microbial community. As an analogy to the process of the well-known ecological succession, we propose the name for this gradual build-up of newly arriving members of the microbiota microbiome succession.

To solve this paradoxical situation we need to examine the gut flora of these human populations. Gut-dwelling bifidobacteria species are typical inhabitants of their intestinal microbiota that have the ability to digest lactose and thus to protect their human hosts from the unintended consequences of lactose intolerance (Masoumi et al., 2021). In an interesting experiment human participants ate parmesan cheese which contains *Bifidobacterium mongoliensis* a typical gut microorganism from dairy cow (Figure 2). The outcome of this study was that these bacteria established themselves in the human gut and divided without complications even weeks later (Milani et al., 2019). We hypothesize that exchange of microbiome between humans and domesticated cow is a process which gradually speeds up after the successful establishment of the first pioneers. This hypothesis is supported by the observation that when participants consumed milk with cheese at the same time, the fixation of the bacteria in the new intestinal environment was more successful (Milani et al., 2019). Based on the well-known processes of the ecological succession, we propose the name for this gradual build-up of newly arriving members of the microbiota microbiome succession.

Domestication, starting more than ten thousand years ago, created new kinds of relationships of humans with animals (Russell, 2022). The closeness to and frequent encounters with domesticated animals has triggered a gradual change in the human microbiome that was not merely quantitative but also qualitative.

There is published evidence that many bacteria in the human gut produce essential vitamins like B12 (Degnan et al., 2014). There are human intestinal worms that make use of the products of the gut-colonising microbiota and take up these vitamins causing B12 deficiency-related diseases in the human host (Sharma et al., 2018). Existing evidence from *Caenorhabditis elegans* indicates that these vitamins are essential for the normal function of these nematodes (Bito et al., 2013) indicating the dependency of the appearance of one community onto the existence of another as seen in ecological succession.

An interesting comparative gut metagenome analysis has shown that carbohydrate active enzymes are frequent in the Japanese population and that they are absent in metagenome data from North American individuals (Hehemann et al., 2010). Seaweeds make an important contribution to the daily diet in Japan, and marine red algae *Porphyra* spp. (nori) is the most important nutritional seaweed, traditionally used to prepare sushi. This indicates that seaweeds with associated marine bacteria may have been the route by which these novel carbohydrate active enzymes were acquired in human gut bacteria, and that contact with non-sterile food may be a general factor in carbohydrate active enzymes diversity in human gut microbes. This exemplifies how new microorganisms can enter and colonize gut microbiota and open the opportunity for horizontal gene transfer and microbiome succession.

Finally, we would like to draw the attention to an intriguing observation that might contribute to our understanding of the above phenomena. A recent large-scale study using more than 9,700 human metagenomes and computational strain-level profiling showed exchange of microbiome in cohabiting humans (Valles-Colomer et al., 2023). They found substantial bacterial strain sharing among cohabiting individuals, with 12 and 32% median strain-sharing rates for the gut and oral microbiomes. Interestingly, they also found that time since cohabitation affected strain sharing more than age or genetics did. There is anecdotical evidence that married couples may phenotypically become more similar to each other after decades of living together. To date this observation has had no convincing proof and there has been also a lack of a theory giving a plausible explanation to the phenomenon (Tea-makorn and Kosinski, 2020). Our hypothesis about the role of microbiome exchange in phenotypical traits (including diseases and digestive traits) might be the reason behind this surprising observation.

## Microbiome succession is facilitated by bacterial conjugation and transfer of phenotypes

Not only degree of exchange of microbial cells is important in the gradual facilitation of the process of microbiome succession but molecular genetic mechanisms like bacterial conjugation can also play a role. This process is mediated by extrachromosomal circular DNA called plasmids. Plasmids are horizontally transmitted from a donor cell to a recipient through a molecular structure named pilus (Koraimann and Wagner, 2014). This is a medically significant mechanism because bacteria reportedly acquire new virulence and antibiotic resistance genes through this way (Gal-Mor and Finlay, 2006). We propose that this process is also important in pathogen emergence. During the process of microbiome succession, virulence genes of the newcomers are being horizontally spread, modifying the biochemical environment to be more comfortable for new pathogenic microbes. A recent experimental research has indicated that not only virulence and antibiotic resistance genes can spread through bacterial conjugation but B12 vitamin mobilization ability too (Frye et al., 2021). As mentioned earlier this might directly facilitate establishment of parasitic helminths in the host’s intestinal system (Mejia et al., 2020).

There are further examples for members of the microbiome encoding important phenotypic traits independently from the so called nuclear genes of the host organism. Experiments with mice where the intestinal microbiota was fully cleared from the gut and replaced by new one shows that diverse medical conditions like obesity, colorectal cancer and diabetes can be transmitted from an affected donor to a previously unaffected recipient by transplantation of the microbiome (Bäckhed et al., 2004; Vrieze et al., 2012; Hu et al., 2013; Poutahidis et al., 2015). More importantly, phenotype transmission experiments not only work between members of the same species but for different species too. Obese people’s faecal transplantation to lean mice can transmit the human phenotype (Fei and Zhao, 2013).

As mentioned earlier, the microbiome of a given host has a protective role to prevent the invasion of potentially harmful foreign microorganisms in the form of colonization resistance (Pickard and Núñez, 2019). In human medical practice after the patients were treated with wide spectrum antibiotics the microbiome vanishes and they become susceptible to the aggressive and dangerous gut pathogen *Clostridium difficile*. The most effective treatment of this condition is the faecal transplantation from a healthy human donor (Juul et al., 2018). Other experiments indicate that upon infection of a resistant host animal, through the replacement of its microbiome with another, susceptible mouse’s microbiome, these animals can be effectively infected by the pathogen too (Willing et al., 2011). This confirms our main hypothesis that the process of gradual and mutual microbiome succession between host species might facilitate the appearance of emerging infectious diseases.

## Infection of the host with one pathogen can open the door for subsequent ones

Although the above mentioned mechanisms seem to be constant and gradual processes, they are not. There are many independent influencing factors which can increase the chance and frequency of spillover in a given time window. We think that one of the most important one is the presence of seasonal infections like influenza or a situation when a pandemic has broken out like the recent COVID-19 disaster. When the host’s immunological ability is heavily loaded by one pathogen, this situation can open the door for another one (Devi et al., 2021). Pathogens can induce systemic immune change tending to cause decreased immunity and an increased risk of secondary infections. In the case of seasonal influenza the co-occurrence of secondary bacterial infections and diseases like pneumonia or otitis media are very high (Morris et al., 2017). Sadly, there were a lot of cases of mucormycosis, an invasive fungal infection and sepsis in COVID-19 patients with serious symptoms (Abumayyaleh et al., 2021; Aranjani et al., 2021).

We believe that these examples are not exceptions but indicators of a general rule which can easily be applied to the case of emergent pathogens or the between-species transmission of non-pathogenic microbiota. The probable mechanism behind this phenomenon is immunosuppression by an ongoing infection that is another independent factor facilitating the process of microbiome succession and thus the route to spillover.

## How can we test these hypotheses?

Undoubtedly the main limitation of our hypothesis is that it lacks direct experimental evidence. There are new metagenomic techniques by which we can follow the spreading of antibiotic resistance genes even within large distances (i.e., multiple hospitals scattered around in different states of the US) and in a wide community of bacterial species (Salamzade et al., 2022). We can apply similar whole-genome sequence comparison method to follow virulence genes with invasive capabilities which makes the jump to new host species possible. Moreover, we can artificially insert the mentioned virulence genes into different viruses, bacteria and parasites simulating horizontal gene transfer and try to successfully infect new target species in a controlled laboratory environment. In the proposed experiment, we assume that a virulence gene (with a function of invasion of host cells and tissues) can establish itself *via* horizontal gene transfer in a new microorganism’s genome and can modify it to become an emerging infectious agent. We can theoretically test this hypothesis by having two host organisms from different species namely Species A and B. Species A has Pathogen A which contains a virulence gene v1. When we insert the gene v1 artificially to a random microorganism of Species B this microorganism becomes Pathogen B and because it harbours v1 it gets the capability to successfully jump to Species A and cause disease in it.

As an alternative, we can also test the hypothesis that the similarity of the microbiome between different host species facilitates the jump of pathogens from one host to another. In such an experiment, we should execute a microbiome transplantation resulting in a microbiome of identical composition in the two different species. By comparing the degree of colonization of the selected pathogen to the control group, we can accept or reject this hypothesis.

## Outlook: The implications of the hypothesis to prevent the next pandemic

As proposed above, the processes of domestication, horizontal gene transfer and microbial succession might be so far neglected important mechanisms behind the many spillover events driven and accelerated by climate change, biodiversity loss and globalization. The recently raging COVID-19 pandemic is teaching us in a painful way that the chapter of emerging infectious diseases cannot be closed by medical scientists alone. To predict which microorganisms and parasites can be dangerous for us in the future is challenging. The DAMA (Document, Assess, Monitor, Act) protocol has been recently suggested as a tool to “anticipate to mitigate” emerging disease (Brooks et al., 2019; Hoberg et al., 2022). Monitoring individual species of potential emerging pathogens, their vectors and reservoirs is undoubtedly necessary (Földvári et al., 2022). Here we propose that chasing dangerous virulence genes and following their circulation in the whole biosphere might be also a targeted and promising strategy for which we already possess the metagenomic toolkits (Salamzade et al., 2022). Moreover, monitoring and assessing the microbiome profile of humans and their domestic and bushmeat animals can be a parallel but another effective way to anticipate and mitigate the harsh consequences of our current pandemic era. Our new hypothesis described here can help watch through the fog of emerging infectious diseases in a new way and provide us an effective proactive weapon to use in the continuous arms race with emerging zoonotic pathogens.

## Data availability statement

The original contributions presented in the study are included in the article/supplementary material, further inquiries can be directed to the corresponding author.

## Author contributions

PA posited the main idea and wrote the original draft. GF helped develop the main idea, contributed in re-drafting and writing the paper, and did substantial reviewing and correction. All authors contributed to the article and approved the submitted version.

## Funding

This work was supported by the National Research, Development and Innovation Office in Hungary (RRF-2.3.1-21-2022-00006), the National Research, Development and Innovation Fund of Hungary (K143622), and the COST Action CA21170 “Prevention, anticipation and mitigation of tick-borne disease risk applying the DAMA protocol (PRAGMATICK).”

## Conflict of interest

The authors declare that the research was conducted in the absence of any commercial or financial relationships that could be construed as a potential conflict of interest.

## Publisher’s note

All claims expressed in this article are solely those of the authors and do not necessarily represent those of their affiliated organizations, or those of the publisher, the editors and the reviewers. Any product that may be evaluated in this article, or claim that may be made by its manufacturer, is not guaranteed or endorsed by the publisher.
